# Vibration Analysis of Functionally Graded Material (FGM) Double-Layered Cabin-like Structure by the Spectro-Geometric Method

**DOI:** 10.3390/ma18061231

**Published:** 2025-03-10

**Authors:** Dongze He, Rui Zhong, Qingshan Wang, Bin Qin

**Affiliations:** 1 School of Mechanical Engineering, Yanshan University, Qinhuangdao 066004, China; 2 Hebei Innovation Center for Equipment Lightweight Design and Manufacturing, Yanshan University, Qinhuangdao 066004, China; 3 College of Mechanical and Electrical Engineering, Central South University, Changsha 410083, China; 4 State Key Laboratory of Precision Manufacturing for Extreme Service Performance, Central South University, Changsha 410083, China; 5 Key Laboratory of Traffic Safety on Track, Ministry of Education, School of Traffic & Transportation Engineering, Central South University, Changsha 410075, China

**Keywords:** spectral geometry method, FGM cylindrical double-walled shell with internal structure, artificial virtual spring approach, free vibration, steady response analysis

## Abstract

This study presents a spectro-geometric vibration model for analyzing free as well as forced vibration properties for FGM cylindrical double-walled shells with internal structures. The boundary conditions and coupling effects are modeled using an artificial virtual spring approach, which allows for the simulation of arbitrary boundary and coupling conditions by varying the elastic spring stiffness coefficients. The spectral geometry method is employed to represent the displacement variables of the FGM substructure, overcoming the discontinuity phenomenon commonly observed when traditional Fourier series are used. The dynamic equations of the FGM cylindrical double-walled shell with an internal structure are derived using the first-order shear deformation assumption and the Rayleigh–Ritz method, and the corresponding vibration solutions are computed. The model’s reliability and prediction accuracy are confirmed through convergence checks and numerical comparisons. Additionally, parametric studies are conducted to examine the influence of material constants, position parameters, and geometric parameters on the shell’s inherent characteristics and steady-state response.

## 1. Introduction

Functionally graded material (FGM) is a kind of multiphase composite material with continuous changes in macroscopic material properties in space by continuously changing the volume fraction of each constituent material [1,2]. Also, FGMs are advanced composite materials that exhibit a gradual variation in material properties, such as Young’s modulus, Poisson’s ratio, and thermal conductivity, across the thickness of the material. This gradation offers significant advantages in enhancing the structural performance of engineering components, especially in high-stress and thermally demanding environments. FGMs have gained widespread use in various industries, including aerospace, automotive, and marine engineering, due to their ability to optimize material properties for specific applications [1,2]. In particular, underwater vehicles, such as submarines and torpedoes, face significant challenges related to vibration and noise, which can affect their operational efficiency and stealth capabilities. Therefore, the use of FGM cylindrical double-walled shell structures in such applications is a promising approach to address these challenges, as they offer a combination of high mechanical strength, reduced weight, and improved vibration-damping properties [3]. Despite the growing interest in FGM-based structures, there remains a gap in the literature regarding the dynamic behavior of FGM cylindrical shells with internal structures, particularly under complex loading conditions, which this study aims to address.

The importance of vibration analysis has led to the exploration of various materials and structural configurations aimed at enhancing the dynamic response of underwater vehicles [4]. In particular, functionally graded materials (FGMs) have emerged as promising candidates due to their ability to tailor material properties across the thickness of a structure, thereby improving its vibration-damping capabilities. However, while many studies have investigated the dynamic behavior of simple FGM structures, there is limited research on the vibration characteristics of more complex FGM cylindrical shells, particularly those with internal structures. The existing literature largely focuses on linear vibrations in homogeneous materials, with few studies addressing the more complex, real-world scenarios where non-uniform material distributions, internal geometries, and varying boundary conditions come into play [5]. This research aims to bridge this gap by analyzing the free and forced vibration characteristics of FGM cylindrical double-walled shells with internal structures, considering a range of boundary conditions and material gradations.

In the design and development of underwater vehicles, lightweight design and research have become new design development ideas. Because of the excellent performance of composite materials, they have become the main way and technical means in the lightweight design and manufacturing of underwater vehicles. Recent advancements in additive manufacturing processes have provided new methods for modeling complex systems and materials, which may complement the current study on functionally graded materials [6]. These approaches offer insights into the fabrication of graded materials, which could be useful for real-world applications of FGM structures in dynamic environments. Meanwhile, with the maturity and continuous development of material preparation and development technology, functionally graded materials are also widely used in the lightweight design of underwater vehicles. The double-layered cabin-type structure containing an elastic floating raft is capable of simulating the shell structure of underwater submarines, torpedoes, navigators, etc. Compared with the single shell structure, it can better simulate the practical application of engineering. With the gradual development of marine equipment structures to light weight and high speed, its vibration and noise problems are becoming more and more prominent. In the working conditions, the operation of different mechanical equipment will inevitably cause the vibration of the overall equipment structure, causing fatigue damage to the structure and generating acoustic radiation to the outside world, resulting in problems such as acoustic stealth and noise. Therefore, it is of significant theoretical value and technical significance to establish the dynamics analysis model of the double-layered cabin structure containing an elastic floating raft and to analyze and examine its dynamics characteristics.

At present, with many scholars and researchers on shell structure dynamics, research has gradually deepened, producing more shell theories. Classical shell theory [7,8,9,10], first-order shear deformation theory [11,12], and high-order shear deformation theory [13,14,15,16,17] are more widely used and present more scientific research achievements. For the first-order shear deformation theory, Choe et al. [18] obtained the theoretical model of the composite laminated doubly curved revolution shell structures. The investigation of the free vibration behavior of the composite laminated shell structures was discussed using the Jacobi–Ritz method. Kim et al. [19] employed the motion equations of the composite laminated doubly curved revolution shell coupled with the comparison of the multi-segment partitioning and Ritz method. The first-order shear deformation theory was selected to analyze the inherent characteristics of the coupled structure under various boundary conditions. Choe et al. [20] studied the inherent characteristics of FGM doubly curved revolution shell structures. The multilevel partition technique was selected to obtain the numerical model of the FG coupled structure. Also, the displacement variables were assumed in the combination form of Jacobi polynomials and standard Fourier series in the meridional and circumferential directions. Chen et al. [21] were concerned with the dynamic behavior of composite laminated open cylindrical shells and rectangular plates coupled structure. The displacement variables of the shell or plate structures were set in the improved Fourier series. Kim et al. [22] developed the Haar wavelet discretization method to analyze the inherent characteristics of composite laminated shell coupled structure in the inverse form, and the displacement functions were set as the combination of the Haar wavelet series and trigonometric series in the meridional and circumferential direction. Su and Jin [23] analyzed the free vibration characteristics of conical–cylindrical–spherical shell coupled structures, and the Fourier spectral element method was conducted to obtain the mathematics model. In addition, the linear superimposition of Fourier sine functions and nodal Lagrangian polynomials was chosen to assume the movement and rotation variables of shell structures.

For Flügge’s shell theory, Wang et al. [24] focused on the free vibration analysis of different coupled doubly curved revolution shell structures. The Jacobi–Ritz method was selected to obtain the theoretical model of the coupled structures. Chen et al. [25] proposed the wave-based method to analyze the vibration characteristics of the finite cylindrical shell with interior structures, and the Flügge shell theory was adopted to obtain the motion equations for coupled structures. Also, the displacement and rotation variable functions were expanded as the wave function formed. Jia et al. [26] employed the vibration dynamic of ring-stiffened cylindrical shells with inner structures by Flügge shell theory, and the displacement functions were set in the wave functions and Bessel function forms. The artificial spring technique was selected to assume the coupling relationship between the substructures. Yu et al. [27] focused on the acoustic vibration characteristics of ring-stiffened cylindrical shell structure, and the numerical analysis model of the coupled structure was obtained according to the wave propagation method and virtual source method. Qin et al. [28] investigated the free vibration dynamic characteristics of several types of doubly curved shell structures under elastically constrained conditions with the Jacobi–Ritz method. Hu et al. [29] investigated the free vibration characteristics of spring-mass-cylindrical shell coupled structure under simply supported boundary conditions by substructure acceptance method. The calculation correctness was confirmed by the comparison with certain reported references and finite element results.

Furthermore, Dai et al. [30] presented the vibration dynamic characteristics of plate–shell coupled structures with complex boundary conditions, and the displacement variables of the coupled structure were assembled as a two-dimensional improved Fourier series. Hamilton’s principle and Rayleigh–Ritz method were adopted to obtain the characteristic equations of the coupled structure. Zhang et al. [31] developed the semi-analytical method to analyze the free vibration characteristics of annular plate–conical–cylindrical–spherical shell coupled structures with complex boundary conditions. The displacement variables of the substructure were set as the modified Fourier series and auxiliary convergence functions in the generatrix direction and circumferential direction. Combined with the energy variational procedure and Ritz method, the analysis model of the coupled structure was obtained. According to Cao et al. [32], concerning the vibration characteristics of the cylindrical shell–circular plate coupled structure under complex boundary conditions, the coupling springs were arranged at the junction of the coupled structure to assume various coupling relationships. The displacement variables of the shell or plate structure were expressed in the two-dimensional Fourier series, and several supplementary functions, the Rayleigh–Ritz method was conducted to obtain the analysis model. Qin et al. [33] analyzed the vibration characteristics of a rotating cylindrical shell and annular plate coupled structure. The Sanders shell theory and the Mindlin plate theory were adopted to obtain the strain energy of the shell/plate structure. Combined with the Rayleigh–Ritz method, the motion equations of the rotation coupled structure were conducted. Kim et al. [34] presented the Haar wavelet discretization method to investigate the free vibration characteristics of composite laminated conical–cylindrical coupled structures. The displacement variable functions of the cylindrical and conical shell structures were set as the Haar wavelet and integrals forms. Also, the artificial spring technique was selected to achieve the boundary and coupling conditions of the coupled structure. Zhang et al. [35] proposed the vibration dynamic characteristics of circular cylindrical double-shell structures by the improved Fourier series method, and the displacement variables of the cylindrical shell and annular plate structures were conducted as the superposition of standard Fourier series and supplementary functions. Based on the Rayleigh–Ritz, the analytical model of the coupled structure was established. According to Tang et al. [36], concerning the free vibration characteristics of bolted joined cylindrical–cylindrical shell structure by the Sanders’ shell theory, the motion equations of the structure of the couple were obtained by the coupling stiffness of the connection condition. Pan and Zhang [37] developed the acoustic-structure coupling analysis model of a submerged double cylindrical shell with stringers, rings, and annular plates coupled structure. The symplectic duality system combined with the Hamiltonian function was adopted to obtain the vibration and acoustic responses of the cylindrical shell and plate coupled structure.

The existing literature indicates that significant progress has been made in studying the dynamic characteristics of FGM plates, shells, and plate–shell coupled structures. However, there is a lack of research addressing the vibration characteristics of FGM cylindrical double-walled shells with internal structures under elastic coupling conditions. Developing a dynamic model for such a structure requires consideration of the coupling mechanisms between various plate and shell components, such as the coupling conditions between rectangular or annular plates and cylindrical shells, as well as the simulation of an elastic floating raft structure. Consequently, establishing an accurate dynamic model for the FGM cylindrical double-walled shell with internal structure and performing its dynamic characteristic analysis presents a significant challenge.

## 2. Theoretical Modeling

The scope of this study is focused on the vibration characteristics model of FGM coupled structure by the spectral geometry method with the first-order shear deformation theory. The displacement variables of the shell/plate substructure are described uniformly as a modified triangular series of a spectral form. According to the Rayleigh–Ritz method, the dynamic equation of the FGM double-layered coupled structure is derived. The convergence and accuracy of the presented analyzed model are verified by the numerical examples and the comparison with the results by the finite element method. Furthermore, the effect of material constant, position variables, and geometric parameters on the natural frequencies and steady response characteristics of FGM cylindrical double-walled shell with internal structure with classical and elastic boundary conditions are discussed. The main purpose of this paper is to establish a numerical analysis model of underwater vehicle cabin class structure, to explore the effect of different parameters, and to provide some theoretical basis for the design and development of vibration damping of the underwater vehicle structure. Given the importance of understanding the dynamic behavior of FGM double-layered cabin-like structures, the next step is to model these structures under various boundary conditions, as outlined in the following methodology section.

**Figure 1:**
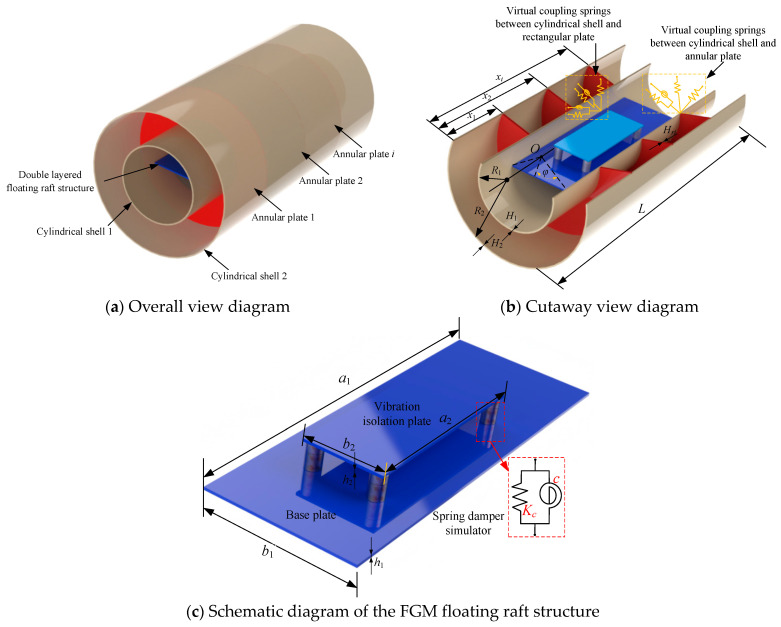
Schematic diagram of the FGM cylindrical double-walled shell with internal structure.

The FGM cylindrical double-walled shell with internal structure is displayed in Figure 1. The whole FGM structure consists of the inner cylindrical shell 1, the outer cylindrical shell 2, the annular plate *i,* and the double-layered floating raft structure. It should be noted that the entire structure is composed of FGM material, and the material properties remain consistent. Furthermore, this study relies on several key assumptions to simplify the modeling of the FGM cylindrical double-walled shell structure and to facilitate the analytical treatment of its dynamic behavior. The following assumptions were made:

- Linear Material Behavior: It is assumed that the material behavior of the FGM is linear-elastic throughout the analysis;
- Perfect Functionally Graded Materials (FGMs): The model assumes a perfect FGM, meaning that the material properties (e.g., Young’s modulus and Poisson’s ratio) vary smoothly across the thickness of the shell;
- Neglect of Nonlinear Vibrations and Imperfections: The study assumes linear free and forced vibrations without considering nonlinear effects that may arise from large deflections or material imperfections.

In Figure 1b, the schematic cutaway view diagram of the FGM cylindrical double-walled shell with internal structure is shown to represent the connectivity of the substructures as well as the partial geometrical parameters. The length of double-layered cylindrical shells 1 and 2 is defined as *L*. Also, *R*_1_ and *R*_2_ represent the radius of the cylindrical shells 1 and 2, and the thicknesses of shells 1 and 2 are shown as *H*_1_ and *H*_2_. The location of the annular plate *i* is defined as *x_i,_* which is located in the axial direction, the thickness of the annular plate is defined as *H_ri_*, and the number of annular plates is set as *N_an_*. Also, the vibration isolation plate and the base plate are connected by four sets of spring damper simulators to form the FGM double-layered floating raft structure, and the specific structural form is shown in Figure 1c. The length, width, and thickness of the base foundation rectangular plate are set as *a*_1_, *b*_1,_ and *h*_1_. Furthermore, *a*_2_, *b*_2,_ and *h*_2_ represent the geometric parameters of the vibration isolation plate. *K_c_* and *c* present the linear spring stiffness and damping parameters of the damper simulator.

Between the connection edge of the cylindrical shell–annular plate and the cylindrical shell–rectangular plate, the virtual coupling spring technical is used to simulate the coupling connection form between various substructures. Furthermore, the material properties, including Young’s modulus, Poisson’s ratio, and mass density of the FGM shell/plate structure, are defined as *E*_1_, *ν*_1_, *ρ*_1,_ and *E*_2_, *ν*_2_, *ρ*_2_. So, the material constant of FGM is defined as follows:

(1) $$E\left(z\right)=\left(E_{1}-E_{2}\right)V+E_{2}$$

(2) $$\nu \left(z\right)=\left(\nu_{1}-\nu_{2}\right)V+\nu_{2}$$

(3) $$\rho \left(z\right)=\left(\rho_{1}-\rho_{2}\right)V+\rho_{2}$$

where *V* is the volume fraction of the FGM material, as

(4) $$V={\left[1-a\left(\frac{1}{2}+\frac{z}{h}\right)+b{\left(\frac{1}{2}+\frac{z}{h}\right)}^{c}\right]}^{p}$$

in which *p* is the power law exponent, *a*, *b,* and *c* are material variation profiles, and *h* represents the thickness in the *z*-direction. It should be emphasized that the FGM material is assumed to be the ideal case in this paper. However, it is unavoidable that there are imperfections in FGM material compositions that take defects into account [38].

### 2.1. Admissible Functions of Displacement Variables

The admissible functions of the displacement and rotation variables for the FGM shell/plate structure are uniformly described as an improved triangular series in spectral form, as

(5) $$\begin{array}{c} u_{0,q}(\alpha ,\beta ,t)=\sum_{m=-2}^{\infty }\sum_{n=-2}^{\infty }U_{n,q}\left(t\right)\Omega_{m,n,q}\left(\alpha ,\beta \right)\\ v_{0,q}(\alpha ,\beta ,t)=\sum_{m=-2}^{\infty }\sum_{n=-2}^{\infty }V_{n,q}\left(t\right)\Omega_{m,n,q}\left(\alpha ,\beta \right)\\ w_{0,q}(\alpha ,\beta ,t)=\sum_{m=-2}^{\infty }\sum_{n=-2}^{\infty }W_{n,q}\left(t\right)\Omega_{m,n,q}\left(\alpha ,\beta \right)\\ \psi_{\alpha ,q}(\alpha ,\beta ,t)=\sum_{m=-2}^{\infty }\sum_{n=-2}^{\infty }\Phi_{n,q}^{\alpha }\left(t\right)\Omega_{m,n,q}\left(\alpha ,\beta \right)\\ \psi_{\beta ,q}(\alpha ,\beta ,t)=\sum_{m=-2}^{\infty }\sum_{n=-2}^{\infty }\Phi_{n,q}^{\beta }\left(t\right)\Omega_{m,n,q}\left(\alpha ,\beta \right) \end{array}$$

where *u*_0,*q*_(*α*, *β*, *t*), *v*_0,*q*_ (*α*, *β*, *t*), and *w*_0,*q*_ (*α*, *β*, *t*) are the variables of displacement from the reference point to the central surface of the plate/shell structure, and *ψ_α_*_,*q*_ (*α*, *β*, *t*) and *ψ_β_*_,*q*_ (*α*, *β*, *t*) are the rotating variables. The subscript *q* = *cy*1, *cy*2, *rec*1, *rec*2, and *ani* correspond to cylindrical shell 1, cylindrical shell 2, base foundation rectangular plate, vibration isolation plate, and *i*th annular plate. Also,

(6) $$\begin{array}{c} U_{n,q}\left(t\right)=\left\{\begin{array}{l} A_{n,q}^{c}e^{j\omega t},n\geq 0\\ A_{n,q}^{s}e^{j\omega t},-2\leq n\leq -1 \end{array}\right.,V_{n,q}\left(t\right)=\left\{\begin{array}{l} B_{n,q}^{c}e^{j\omega t},n\geq 0\\ B_{n,q}^{s}e^{j\omega t},-2\leq n\leq -1 \end{array}\right.\\ W_{n,q}\left(t\right)=\left\{\begin{array}{l} C_{n,q}^{c}e^{j\omega t},n\geq 0\\ C_{n,q}^{s}e^{j\omega t},-2\leq n\leq -1 \end{array}\right.\\ \Phi_{n,q}^{\alpha }\left(t\right)=\left\{\begin{array}{l} D_{n,q}^{c}e^{j\omega t},n\geq 0\\ D_{n,q}^{s}e^{j\omega t},-2\leq n\leq -1 \end{array}\right.,\Phi_{n,q}^{\beta }\left(t\right)=\left\{\begin{array}{l} E_{n,q}^{c}e^{j\omega t},n\geq 0\\ E_{n,q}^{s}e^{j\omega t},-2\leq n\leq -1 \end{array}\right. \end{array}$$

and

(7) $$\Omega_{m,n,q}\left(\alpha ,\beta \right)=\left\{\begin{array}{l} \cos\left(\lambda_{m}\alpha \right)\cos(\lambda_{n}\beta ),\;\mathrm{when}\;n\geq 0\;\mathrm{and}\;m\geq 0\\ \cos\left(\lambda_{m}\alpha \right)\sin(\lambda_{n}\beta ),\;\mathrm{when}\;-2\leq n\leq -1\;\mathrm{and}\;m\geq 0\\ \sin\left(\lambda_{m}\alpha \right)\cos(\lambda_{n}\beta ),\;\mathrm{when}\;n\geq 0\;\mathrm{and}\;-2\leq m\leq -1\\ \sin\left(\lambda_{m}\alpha \right)\sin(\lambda_{n}\beta ),\;\mathrm{when}\;-2\leq n\leq -1\;\mathrm{and}\;-2\leq m\leq -1 \end{array}\right.$$

in which *λ_m_* = *m*π/*L_α_* and *λ_n_* = *n*π/*L_β_*. $A_{n,q}^{c,s}$, $B_{n,q}^{c,s}$, $C_{m,n,q}^{c,s}$, $D_{n,q}^{c,s}$, $E_{n,q}^{c,s}$ are the unknown Fourier series expansion coefficients for the uniform displacement variable functions. Note that, for the cylindrical shell structure, *α* = *x* and *β* = *θ* are used, while *α* = *x*, *β* = *y* for the rectangular plate, and *α* = *x*, *β* = *θ* for the annular plate are considered.

### 2.2. General Equations of Plate/Shell

The FGM cylindrical double-walled shell with internal structure investigated in this paper contains the following three substructures, namely, axisymmetric closed cylindrical shell, annular plate, and rectangular plate structures. Among them, the cylindrical shell and circular plate structure can be regarded as the special case of the open conical shell structure. In other words, when considering the angle of rotation *θ* = 2π and imposing the mechanical continuity conditions at coupled edges (namely, *θ* = 0 and 2π), the open conical shells can be transformed into cylindrical shells and circular plates by setting different cone apex angles respectively. Meanwhile, the rectangular plate structure can be regarded as the special case of the open cylindrical shell. Therefore, through the above analysis, it can be seen that the mechanical equations for the above structures can be obtained by setting different geometrical parameters of open conical shell structure. Therefore, in this section, the FGM open conical shell structure is taken as an example to establish the sub-structural mechanical model in the FGM cylindrical double-walled shell with internal structure.

Following the theory of first-order shear deformation, the detailed formulations for strain and change parameters of the FGM conical shell are defined, as

(8) $$\varepsilon_{x}^{0}=\frac{\partial u}{\partial x}$$

(9) $$\varepsilon_{\theta }^{0}=\frac{1}{R}\frac{\partial v}{\partial \theta }+\frac{u_{0}\sin\alpha_{0}}{R}+\frac{w_{0}\cos\alpha_{0}}{R}$$

(10) $$\varepsilon_{x\theta }^{0}=\frac{\partial v}{\partial x}+\frac{1}{R}\frac{\partial u}{\partial \theta }-\frac{v_{0}\sin\alpha_{0}}{R}$$

(11) $$\chi_{x}=\frac{\partial \phi_{x}}{\partial x}$$

(12) $$\chi_{\theta }=\frac{1}{R}\frac{\partial \phi_{\theta }}{\partial \theta }+\frac{\phi_{x}\sin\alpha_{0}}{R}$$

(13) $$\chi_{x\theta }=\frac{\partial \phi_{\theta }}{\partial x}+\frac{1}{R}\frac{\partial \phi_{x}}{\partial \theta }-\frac{\phi_{\theta }\sin\alpha_{0}}{R}$$

where *R* = *x*sin*α*_0_. For the FGM cylindrical shell structure, *R* = *R*_0_ is a constant; when *α*_0_ = π/2, it will be transformed into a circular plate structure. At the same time, when the radius of the FGM cylindrical shell is set to infinity and the smaller angle is taken, it is transformed into the FGM rectangular plate structure. It is possible to obtain the equations of the strain–deformation relationship of the FGM conical shell structure by Hook’s law, as

(14) $$\left[\begin{array}{c} \sigma_{x}\\ \sigma_{\theta }\\ \tau_{x\theta }\\ \tau_{\theta z}\\ \tau_{xz} \end{array}\right]=\left[\begin{array}{ccccc} Q_{11}\left(z\right) & Q_{12}\left(z\right) & 0 & 0 & 0\\ Q_{12}\left(z\right) & Q_{11}\left(z\right) & 0 & 0 & 0\\ 0 & 0 & Q_{66}\left(z\right) & 0 & 0\\ 0 & 0 & 0 & Q_{66}\left(z\right) & 0\\ 0 & 0 & 0 & 0 & Q_{66}\left(z\right) \end{array}\right]\left[\begin{array}{c} \varepsilon_{x}\\ \varepsilon_{\theta }\\ \gamma_{x\theta }\\ \gamma_{xz}\\ \gamma_{\theta z} \end{array}\right]$$

where *Q*_11_(*z*), *Q*_12,_(*z*), and *Q*_16_(*z*) are the elastic stiffness constant of the FGM conical shell, which is defined by material parameters *E*(*z*) and *ν*(*z*) of the FGM, as

(15) $$Q_{11}\left(z\right)=\frac{E\left(z\right)}{1-\nu^{2}\left(z\right)},\;Q_{12}\left(z\right)=\frac{\nu \left(z\right)E\left(z\right)}{1-\nu^{2}\left(z\right)},\;Q_{66}\left(z\right)=\frac{E\left(z\right)}{2\left[1+\nu \left(z\right)\right]}$$

By integrating the constraint variables across the thickness, the constitutive equations of the FGM conical shell structure are obtained

(16) $$\left\{\begin{array}{c} N_{x}\\ N_{\theta }\\ N_{x\theta } \end{array}\right\}=\left[\begin{array}{ccc} A_{11} & A_{12} & 0\\ A_{12} & A_{11} & 0\\ 0 & 0 & A_{66} \end{array}\right]\left\{\begin{array}{c} \varepsilon_{x}^{0}\\ \varepsilon_{\theta }^{0}\\ \gamma_{x\theta }^{0} \end{array}\right\}+\left[\begin{array}{ccc} B_{11} & B_{12} & 0\\ B_{12} & B_{11} & 0\\ 0 & 0 & B_{66} \end{array}\right]\left\{\begin{array}{c} \chi_{x}\\ \chi_{\theta }\\ \chi_{x\theta } \end{array}\right\}$$

(17) $$\left\{\begin{array}{c} M_{x}\\ M_{\theta }\\ M_{x\theta } \end{array}\right\}=\left[\begin{array}{ccc} B_{11} & B_{12} & 0\\ B_{12} & B_{11} & 0\\ 0 & 0 & B_{66} \end{array}\right]\left\{\begin{array}{c} \varepsilon_{x}^{0}\\ \varepsilon_{\theta }^{0}\\ \gamma_{x\theta }^{0} \end{array}\right\}+\left[\begin{array}{ccc} D_{11} & D_{12} & 0\\ D_{12} & D_{11} & 0\\ 0 & 0 & D_{66} \end{array}\right]\left\{\begin{array}{c} \chi_{x}\\ \chi_{\theta }\\ \chi_{x\theta } \end{array}\right\}$$

(18) $$\left\{\begin{array}{c} Q_{\theta }\\ Q_{x} \end{array}\right\}=K_{c}\left[\begin{array}{cc} A_{66} & 0\\ 0 & A_{66} \end{array}\right]\left\{\begin{array}{c} \gamma_{\theta z}\\ \gamma_{xz} \end{array}\right\}$$

where *K_c_* means the shear correction factor, as 5/6. *A*_11_, *A*_12_, and *A*_66_ are the extensional stiffness coefficients, as

(19) $$A_{11}=\int_{-h/2}^{h/2}Q_{11}\left(z\right)dz,A_{12}=\int_{-h/2}^{h/2}Q_{12}\left(z\right)dz,A_{66}=\int_{-h/2}^{h/2}Q_{66}\left(z\right)dz$$

*B*_11_, *B*_12_, and *B*_66_ are the extensional-bending coupling stiffness coefficients, as

(20) $$B_{11}=\int_{-h/2}^{h/2}Q_{11}\left(z\right)zdz,B_{12}=\int_{-h/2}^{h/2}Q_{12}\left(z\right)zdz,B_{66}=\int_{-h/2}^{h/2}Q_{66}\left(z\right)zdz$$

*D*_11_, *D*_12_, and *D*_66_ are the bending coupling stiffness coefficients, as

(21) $$D_{11}=\int_{-h/2}^{h/2}Q_{11}\left(z\right)z^{2}dz,D_{12}=\int_{-h/2}^{h/2}Q_{12}\left(z\right)z^{2}dz,D_{66}=\int_{-h/2}^{h/2}Q_{66}\left(z\right)z^{2}dz$$

In this paper, the spectral geometry method with the Ritz method is proposed to define the vibration analysis model of an FGM cylindrical double-walled shell with an internal structure. The energy expressions of the FGM coupled structure, which include the total potential energy and total kinetic energy, can be obtained. The strain energy of the FGM conical shell structure is shown as

(22) $$U_{v}=\frac{1}{2}\iint_{S}\left(\begin{array}{l} N_{x}\varepsilon_{x}^{0}+N_{\theta }\varepsilon_{\theta }^{0}+N_{x\theta }\varepsilon_{x\theta }^{0}+M_{x}\chi_{x}+M_{\theta }\chi_{\theta }\\ +M_{x\theta }\chi_{x\theta }+Q_{x}\gamma_{xz}^{0}+Q_{\theta }\gamma_{\theta z}^{0} \end{array}\right)x\sin\alpha_{0}dxd\theta$$

The kinetic energy *T* for the FGM conical shell structure is defined as

(23) $$T=\frac{1}{2}\iint \left\{\begin{array}{l} I_{0}{\left(\frac{\partial u_{0}}{\partial t}\right)}^{2}+I_{0}{\left(\frac{\partial v_{0}}{\partial t}\right)}^{2}+I_{0}{\left(\frac{\partial w_{0}}{\partial t}\right)}^{2}+I_{2}{\left(\frac{\partial \varphi_{\alpha }}{\partial t}\right)}^{2}+I_{2}{\left(\frac{\partial \varphi_{\beta }}{\partial t}\right)}^{2}\\ +2I_{1}\left(\frac{\partial u_{0}}{\partial t}\right)\left(\frac{\partial \varphi_{\alpha }}{\partial t}\right)+2I_{1}\left(\frac{\partial v_{0}}{\partial t}\right)\left(\frac{\partial \varphi_{\beta }}{\partial t}\right) \end{array}\right\}ABd\alpha d\beta$$

where *I*_0_, *I*_1_, and *I*_2_ are the inertia mass coefficients for the FGM conical shell structures, as

(24) $$I_{0}=\int_{-h/2}^{h/2}\rho \left(z\right)dz,I_{1}=\int_{-h/2}^{h/2}\rho \left(z\right)zdz,I_{2}=\int_{-h/2}^{h/2}\rho \left(z\right)z^{2}dz$$

### 2.3. Continuity and General Boundary Conditions

For FGM closed cylindrical shell structures, the kinematic and physical continuity conditions need to be satisfied at the coupling boundary at *θ* = 0 and *θ* = 2π. At the continuous boundary, the continuity condition for the displacement can be expressed as

(25) $$\begin{array}{l} {\left.u_{0,cyj}\right|}_{\theta =0}={\left.u_{0,cyj}\right|}_{\theta =2\pi }\\ {\left.v_{0,cyj}\right|}_{\theta =0}={\left.v_{0,cyj}\right|}_{\theta =2\pi }\\ {\left.w_{0,cyj}\right|}_{\theta =0}={\left.w_{0,cyj}\right|}_{\theta =2\pi }\\ {\left.\varphi_{x,cyj}\right|}_{\theta =0}={\left.\varphi_{x,cyj}\right|}_{\theta =2\pi }\\ {\left.\varphi_{\theta ,cyj}\right|}_{\theta =0}={\left.\varphi_{\theta ,cyj}\right|}_{\theta =2\pi } \end{array}$$

**Figure 2:**
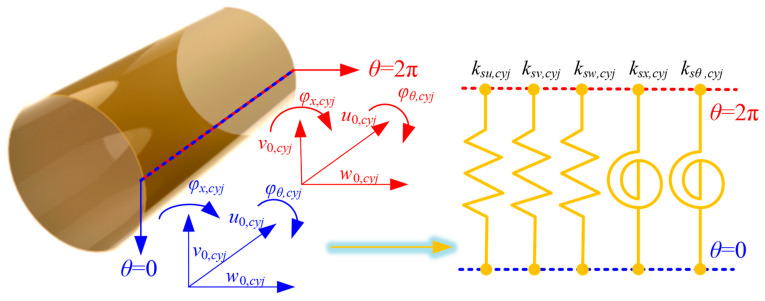
Coupling effect model at the coupling boundary of FGM cylindrical shells.

In this paper, by integrating the artificial virtual coupling technology, the corresponding continuity conditions can be satisfied by the corresponding coupling springs, which can meet the corresponding requirements, as shown in Figure 2.

Furthermore, the coupling equations at the continuity condition of the FGM cylindrical shell can be expressed, respectively, as follows:

(26) $$\begin{array}{l} k_{su,cyj}\left({\left.u_{0,cyj}\right|}_{\theta =0}-{\left.u_{0,cyj}\right|}_{\theta =2\pi }\right)={\left.N_{x,cyj}\right|}_{\theta =0}\\ k_{sv,cyj}\left({\left.v_{0,cyj}\right|}_{\theta =0}-{\left.v_{0,cyj}\right|}_{\theta =2\pi }\right)={\left.N_{\theta ,cyj}\right|}_{\theta =0}\\ k_{sw,cyj}\left({\left.w_{0,cyj}\right|}_{\theta =0}-{\left.w_{0,cyj}\right|}_{\theta =2\pi }\right)={\left.M_{x,cyj}\right|}_{\theta =0}\\ k_{sx,cyj}\left({\left.\varphi_{x,cyj}\right|}_{\theta =0}-{\left.\varphi_{x,cyj}\right|}_{\theta =2\pi }\right)={\left.Q_{x,cyj}\right|}_{\theta =0}\\ k_{s\theta ,cyj}\left({\left.\varphi_{\theta ,cyj}\right|}_{\theta =0}-{\left.\varphi_{\theta ,cyj}\right|}_{\theta =2\pi }\right)={\left.Q_{\theta ,cyj}\right|}_{\theta =0} \end{array}$$

where *k_su_*_/*v*/*w*/*x*/*θ*,*cyj*_ are the spring stiffness in various directions at the coupling edge. In addition, for cylindrical shell *j* (*j* = 1 and 2), the coupling potential energy *U_s_*,*_cyj_* between *θ* = 0 and *θ* = 2π needs to be considered based on the virtual spring technology. So, the elastic potential energy of the coupling potential energy is shown as

(27) $$U_{s,cyj}=\frac{1}{2}\int_{-H_{j}/2}^{H_{j}/2}\int_{0}^{L}\left[\begin{array}{l} k_{su,cyj}{\left({\left.u_{0,cyj}\right|}_{\theta =0}-{\left.u_{0,cyj}\right|}_{\theta =2\pi }\right)}^{2}+k_{sv,cyj}{\left({\left.v_{0,cyj}\right|}_{\theta =0}-{\left.v_{0,cyj}\right|}_{\theta =2\pi }\right)}^{2}\\ +k_{sw,cyj}{\left({\left.w_{0,cyj}\right|}_{\theta =0}-{\left.w_{0,cyj}\right|}_{\theta =2\pi }\right)}^{2}+k_{sx,cyj}{\left({\left.\varphi_{x,cyj}\right|}_{\theta =0}-{\left.\varphi_{x,cyj}\right|}_{\theta =2\pi }\right)}^{2}\\ +k_{s\theta ,cyj}{\left({\left.\varphi_{\theta ,cyj}\right|}_{\theta =0}-{\left.\varphi_{\theta ,cyj}\right|}_{\theta =2\pi }\right)}^{2} \end{array}\right]dxdz$$

Furthermore, for annular plate *i* (*i* = 1 − *N_a_*), the coupling potential energy *U_s_*,*_ani_* between *θ* = 0 and *θ* = 2π needs to be considered based on the virtual spring technology. So, the elastic potential energy of the coupling potential energy *U_s_*,*_ani_* is shown as

(28) $$U_{s,ani}=\frac{1}{2}\int_{-H_{ri}/2}^{H_{ri}/2}\int_{0}^{R_{2}-R_{1}}\left[\begin{array}{l} k_{su,ani}{\left({\left.u_{0,ani}\right|}_{\theta =0}-{\left.u_{0,cyj}\right|}_{\theta =2\pi }\right)}^{2}+k_{sv,ani}{\left({\left.v_{0,ani}\right|}_{\theta =0}-{\left.v_{0,ani}\right|}_{\theta =2\pi }\right)}^{2}\\ +k_{sw,ani}{\left({\left.w_{0,ani}\right|}_{\theta =0}-{\left.w_{0,ani}\right|}_{\theta =2\pi }\right)}^{2}+k_{sx,ani}{\left({\left.\varphi_{x,ani}\right|}_{\theta =0}-{\left.\varphi_{x,ani}\right|}_{\theta =2\pi }\right)}^{2}\\ +k_{s\theta ,ani}{\left({\left.\varphi_{\theta ,ani}\right|}_{\theta =0}-{\left.\varphi_{\theta ,ani}\right|}_{\theta =2\pi }\right)}^{2} \end{array}\right]dxdz$$

Also, the connection relationships of the cylindrical shell and rectangular plate at edges *y* = 0 and *y* = *b*_1_ need to be considered, as

(29) $$\left\{\begin{array}{c} u_{0,rec}\\ v_{0,rec}\\ w_{0,rec}\\ \varphi_{y,rec} \end{array}\right\}=\left[\begin{array}{cccc} 1 & 0 & 0 & 0\\ 0 & \cos\varphi & \sin\varphi & 0\\ 0 & -\sin\varphi & \cos\varphi & 0\\ 0 & 0 & 0 & 1 \end{array}\right]\left\{\begin{array}{c} u_{0,cy}\\ v_{0,cy}\\ w_{0,cy}\\ \varphi_{\theta ,cy} \end{array}\right\},y=0,\theta =90^{\circ }+\varphi$$

(30) $$\left\{\begin{array}{c} u_{0,rec}\\ v_{0,rec}\\ w_{0,rec}\\ \varphi_{y,rec} \end{array}\right\}=\left[\begin{array}{cccc} 1 & 0 & 0 & 0\\ 0 & \cos\varphi & -\sin\varphi & 0\\ 0 & \sin\varphi & \cos\varphi & 0\\ 0 & 0 & 0 & 1 \end{array}\right]\left\{\begin{array}{c} u_{0,cy}\\ v_{0,cy}\\ w_{0,cy}\\ \varphi_{\theta ,cy} \end{array}\right\},y=b_{1},\theta =90^{\circ }-\varphi$$

**Figure 3:**
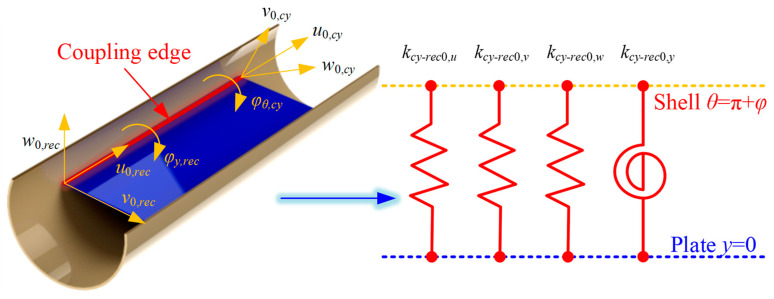
Coupling effect model at the coupling edge *θ* = π + *φ* and *y* = 0 of FGM cylindrical shell and rectangular plate.

Similar to the FGM closed cylindrical shell, at the coupling boundary between the FGM cylindrical shell and the rectangular plate, the artificial virtual spring technique is employed to satisfy the corresponding continuity conditions by installing the corresponding coupling springs. In Figure 3, the coupling effect model at the coupling edge *θ* = π + *φ* and *y* = 0 are shown.

Based on this technology, the coupling equations at the connection edge *θ* = π + *φ* and *θ* = π − *φ* of the FGM cylindrical shell and the rectangular plate can be expressed as follows, respectively:

(31) $$\begin{array}{l} k_{cy-rec0,u}\left({\left.u_{0,rec1}\right|}_{y=0}-u_{0,cy1}\right)={\left.N_{x,rec1}\right|}_{y=0}\\ k_{cy-rec0,v}\left({\left.v_{0,rec1}\right|}_{y=0}-v_{0,cy1}\cos\varphi -w_{0,cy1}\sin\varphi \right)={\left.N_{y,rec1}\right|}_{y=0}\\ k_{cy-rec0,w}\left({\left.w_{0,rec1}\right|}_{y=0}-w_{0,cy1}\cos\varphi +v_{0,cy1}\sin\varphi \right)={\left.Q_{x,rec1}\right|}_{y=0}\\ k_{cy-rec0,y}\left({\left.\varphi_{y,rec1}\right|}_{y=0}-\varphi_{\theta ,cy1}\right)={\left.M_{y,rec1}\right|}_{y=0} \end{array}$$

(32) $$\begin{array}{l} k_{cy-rec1,u}\left({\left.u_{0,rec1}\right|}_{y=b_{1}}-u_{0,cy1}\right)={\left.N_{x,rec1}\right|}_{y=b_{1}}\\ k_{cy-rec1,v}\left({\left.v_{0,rec1}\right|}_{y=b_{1}}-v_{0,cy1}\cos\varphi +w_{0,cy1}\sin\varphi \right)={\left.N_{y,rec1}\right|}_{y=b_{1}}\\ k_{cy-rec1,w}\left({\left.w_{0,rec1}\right|}_{y=b_{1}}-w_{0,cy1}\cos\varphi -v_{0,cy1}\sin\varphi \right)={\left.Q_{x,rec1}\right|}_{y=b_{1}}\\ k_{cy-rec1,y}\left({\left.\varphi_{y,rec1}\right|}_{y=b_{1}}-\varphi_{\theta ,cy1}\right)={\left.M_{y,rec1}\right|}_{y=b_{1}} \end{array}$$

where *k_cy_*_-*rec*0/1, *u*/*v*/*w*/*y*_ are the linear and rotating elastic springs at the coupling edges. Also, the elastic coupling potential energy of cylindrical shell 1 and rectangular plate is conducted as

(33) $$\begin{array}{ll} U_{cy-rec} & =\frac{1}{2}\int_{-h_{1}/2}^{h_{1}/2}\int_{0}^{a_{1}}\left[\begin{array}{l} k_{cy-rec0,u}{\left({\left.u_{0,rec1}\right|}_{y=0}-u_{0,cy1}\right)}^{2}\\ +k_{cy-rec0,v}{\left({\left.v_{0,rec1}\right|}_{y=0}-v_{0,cy1}\cos\varphi -w_{0,cy1}\sin\varphi \right)}^{2}\\ +k_{cy-rec0,w}{\left({\left.w_{0,rec1}\right|}_{y=0}-w_{0,cy1}\cos\varphi +v_{0,cy1}\sin\varphi \right)}^{2}\\ +k_{cy-rec0,y}{\left({\left.\varphi_{y,rec1}\right|}_{y=0}-\varphi_{\theta ,cy1}\right)}^{2} \end{array}\right]dxdz\\ & +\frac{1}{2}\int_{-h_{1}/2}^{h_{1}/2}\int_{0}^{a_{1}}\left[\begin{array}{l} k_{cy-rec1,u}{\left({\left.u_{0,rec1}\right|}_{y=b_{1}}-u_{0,cy1}\right)}^{2}\\ +k_{cy-rec1,v}{\left({\left.v_{0,rec1}\right|}_{y=b_{1}}-v_{0,cy1}\cos\varphi +w_{0,cy1}\sin\varphi \right)}^{2}\\ +k_{cy-rec1,w}{\left({\left.w_{0,rec1}\right|}_{y=b_{1}}-w_{0,cy1}\cos\varphi -v_{0,cy1}\sin\varphi \right)}^{2}\\ +k_{cy-rec1,y}{\left({\left.\varphi_{y,rec1}\right|}_{y=b_{1}}-\varphi_{\theta ,cy1}\right)}^{2} \end{array}\right]dxdz \end{array}$$

For the FGM cylindrical double-walled shell with internal structure in this article, the connection relationship between the cylindrical shell and annular plate should be considered. For the FGM coupled structure investigated in this paper, the coupling connection relationship between the FGM cylindrical shell and the circular plate needs to be considered in two cases. That is, the continuity conditions of the circular plate and cylindrical shell 1 (inner connection) and the circular plate and cylindrical shell 2 (outer connection) are considered, respectively.

(34) $$\begin{array}{cc} \begin{array}{c} u_{0,cy1}=-w_{0,ani}\\ v_{0,cy1}=v_{0,ani}\\ w_{0,cy1}=u_{0,ani}\\ \varphi_{x,cy1}=\varphi_{x,ani} \end{array} & \begin{array}{c} u_{0,cy2}=-w_{0,ani}\\ v_{0,cy2}=v_{0,ani}\\ w_{0,cy2}=u_{0,ani}\\ \varphi_{x,cy2}=\varphi_{x,ani} \end{array} \end{array}$$

**Figure 4:**
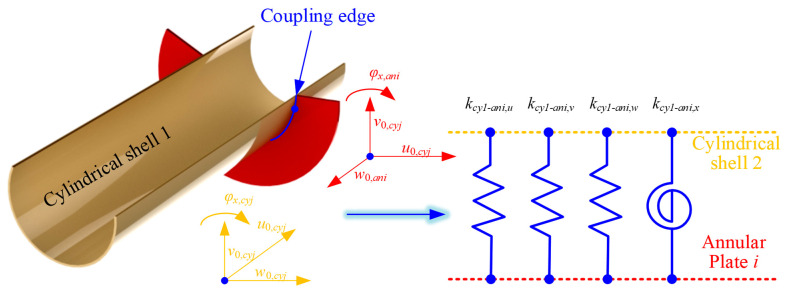
Coupling effect model at the coupling edge of FGM annular plate and cylindrical shell (inner).

**Figure 5:**
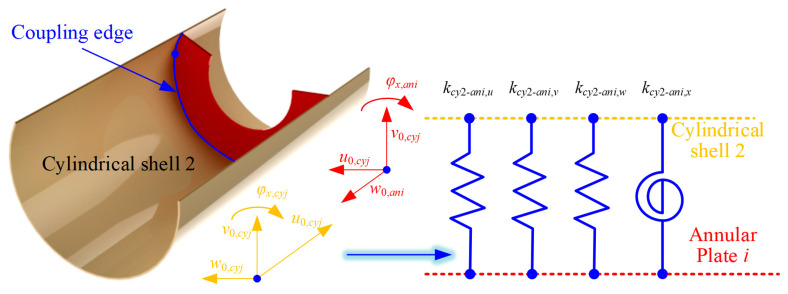
Coupling effect model at the coupling edge of FGM annular plate and cylindrical shell (outer).

The artificial virtual spring technique is used to express the continuity conditions for the two kinds of distinct conditions as follows:

(35) $$\begin{array}{c} k_{cy1-ani,u}\left(u_{0,cy1}+w_{0,ani}\right)=N_{x,cy1}\\ k_{cy1-ani,v}\left(v_{0,cy1}-v_{0,ani}\right)=N_{\theta ,cy1}\\ k_{cy1-ani,w}\left(w_{0,cy1}-u_{0,ani}\right)=Q_{x,cy1}\\ k_{cy1-ani,x}\left(\varphi_{x,cy1}-\varphi_{x,ani}\right)=M_{x,cy1} \end{array}$$

(36) $$\begin{array}{c} k_{cy2-ani,u}\left(u_{0,cy2}+w_{0,ani}\right)=N_{x,cy2}\\ k_{cy2-ani,v}\left(v_{0,cy2}-v_{0,ani}\right)=N_{\theta ,cy2}\\ k_{cy2-ani,w}\left(w_{0,cy2}-u_{0,ani}\right)=Q_{x,cy2}\\ k_{cy2-ani,x}\left(\varphi_{x,cy2}-\varphi_{x,ani}\right)=M_{x,cy2} \end{array}$$

where *k_cy_*_1/2-*ani*, *u*/*v*/*w*/*x*_ are the linear and rotating elastic springs at the coupling edges. Also, in Figure 4 and Figure 5, the representation of the coupling effect model at the coupling edge of the FGM annular plate and cylindrical shell is shown.

Furthermore, the potential energy of the FGM cylindrical double-walled shell with internal structure and the strain energy of the FGM shell/plate substructure with virtual coupling spring technology should be proposed. The elastic coupling potential energy of cylindrical shell 1/2 and annular plate *i* is defined as

(37) $$\begin{array}{ll} U_{cy-ani} & =\frac{1}{2}{\int_{0}^{2\pi }\int_{-H_{ri}/2}^{H_{ri}/2}\left[\begin{array}{l} k_{cy1-ani,u}{\left(u_{0,cy1}+w_{0,ani}\right)}^{2}+k_{cy1-ani,v}{\left(v_{0,cy}-v_{0,ani}\right)}^{2}+\\ k_{cy1-ani,w}{\left(w_{0,cy1}-u_{0,ani}\right)}^{2}+k_{cy1-ani,x}{\left(\varphi_{x,cy}-\varphi_{x,ani}\right)}^{2} \end{array}\right]}_{x=x_{i}}R_{1}d\theta dz\\ & +\frac{1}{2}\int_{0}^{2\pi }\int_{-H_{ri}/2}^{H_{ri}/2}{\left[\begin{array}{l} k_{cy2-ani,u}{\left(u_{0,cy2}+w_{0,ani}\right)}^{2}+k_{cy2-ani,v}{\left(v_{0,cy}-v_{0,ani}\right)}^{2}+\\ k_{cy2-ani,w}{\left(w_{0,cy2}-u_{0,ani}\right)}^{2}+k_{cy2-ani,x}{\left(\varphi_{x,cy}-\varphi_{x,ani}\right)}^{2} \end{array}\right]}_{x=x_{i}}R_{2}d\theta dz \end{array}$$

Next, similarly to the virtual coupling spring technology, the technology of the virtual boundary spring is selected for simulating boundary conditions of the FGM cylindrical double-walled shell with internal structure in this paper. The linear elastic springs and rotation springs are fixed at two ends of the cylindrical shell structure and the edge *x* = 0 and *x* = *a*_1_ of the base plate.

(38) $$U_{bc-rec}=\frac{1}{2}\int_{0}^{b_{1}}\int_{-h_{1}/2}^{h_{1}/2}\left[\begin{array}{l} {\left(\begin{array}{l} K_{ux,0}{u_{0,rec1}}^{2}+K_{vx,0}{v_{0,rec1}}^{2}+K_{wx,0}{w_{0,rec1}}^{2}\\ +K_{xx,0}{\varphi_{x,rec1}}^{2}+K_{yx,0}{\varphi_{y,rec1}}^{2} \end{array}\right)}_{x=0}\\ +{\left(\begin{array}{l} K_{ux,a}{u_{0,rec1}}^{2}+K_{vx,a}{v_{0,rec1}}^{2}+K_{wx,a}{w_{0,rec1}}^{2}\\ +K_{xx,a}{\varphi_{x,rec1}}^{2}+K_{yx,a}{\varphi_{y,rec1}}^{2} \end{array}\right)}_{x=a_{1}} \end{array}\right]dydz$$

where *K_ux_*_,0/*a*_, *K_vx_*_,0/*a*_, and *K_wx_*_,0/*a*_ are the linear elastic springs, and *K_xx_*_,0/*a*_ and *K_yx_*_,0/*a*_ are the rotation elastic springs for the base plate. Then, the potential energy of the FGM cylindrical shell by the virtual boundary spring technology is shown as

(39) $$U_{bc-cy}=\frac{1}{2}\sum_{j=1}^{2}\left(\begin{array}{l} \int_{-H_{j}/2}^{H_{j}/2}\int_{0}^{2\pi }\left[\begin{array}{l} K_{u0,j}{u_{0,cyj}}^{2}+K_{v0,j}{v_{0,cyj}}^{2}+K_{w0,j}{w_{0,cyj}}^{2}\\ +K_{x0,j}{\varphi_{\alpha ,cyj}}^{2}+K_{\theta 0,j}{\varphi_{\beta ,cyj}}^{2} \end{array}\right]R_{j}d\theta dz\\ +\int_{-H_{j}/2}^{H_{j}/2}\int_{0}^{2\pi }\left[\begin{array}{l} K_{uL,j}{u_{0,cyj}}^{2}+K_{vL,j}{v_{0,cyj}}^{2}+K_{wL,j}{w_{0,cyj}}^{2}\\ +K_{xL,j}{\varphi_{\alpha ,cyj}}^{2}+K_{\theta L,j}{\varphi_{\beta ,cyj}}^{2} \end{array}\right]R_{j}d\theta dz \end{array}\right)$$

where *K_u_*_0/*L,j*_, *K_v_*_0/*L,j*_, and *K_w_*_0/*L,j*_ are the linear elastic springs, *K_x_*_0/*L*,*j,*_ and *K_θ_*_0/*L*,*j*_ are the rotation elastic springs for the FGM cylindrical shell. In this article, the floating raft structure is coupled with cylindrical shell 1 in the FGM cylindrical double-walled shell with internal structure to simulate the cabin structure with a floating raft structure. In the floating raft structure, the spring damping simulator, which includes the elastic supporting springs and damper, is arranged at four connection points. So, the potential energy of the spring damping simulator is shown as

(40) $$U_{c}=\sum_{j=1}^{4}\left\{\begin{array}{l} \frac{1}{2}\left(K_{c,uj}{u_{c,j}}^{2}+K_{c,vj}{v_{c,j}}^{2}+K_{c,wj}{w_{c,j}}^{2}+K_{c,xj}{\varphi_{xc,j}}^{2}+K_{c,yj}{\varphi_{yc,j}}^{2}\right)-\\ \frac{1}{2}c\left({\left(\frac{\partial u_{c,j}}{\partial t}\right)}^{2}+{\left(\frac{\partial v_{c,j}}{\partial t}\right)}^{2}+{\left(\frac{\partial w_{c,j}}{\partial t}\right)}^{2}+{\left(\frac{\partial \varphi_{xc,j}}{\partial t}\right)}^{2}+{\left(\frac{\partial \varphi_{yc,j}}{\partial t}\right)}^{2}\right) \end{array}\right\}$$

where

(41) $$\begin{array}{c} u_{c,j}=u_{0,rec1j}-u_{0,rec2j}\\ v_{c,j}=v_{0,rec1j}-v_{0,rec2j}\\ w_{c,j}=w_{0,rec1j}-w_{0,rec2j}\\ \varphi_{xc,j}=\varphi_{x,rec1j}-\varphi_{x,rec2j}\\ \varphi_{yc,j}=\varphi_{y,rec1j}-\varphi_{y,rec2j} \end{array}$$

In Equation (40), *K_c_*_,*uj*/*vj*/*wj*/*xj*/*yj*_ are the linear and rotation springs of the spring damping simulator and *c* is the damping value. Then, the total elastic potential energy of the FGM cylindrical double-walled shell with internal structure by the virtual coupling spring technology, virtual boundary spring technology, and spring damping simulator is shown as

(42) $$U_{total}=U_{cy-an}+U_{cy-rec}+U_{bc-cy}+U_{bc-rec}+U_{c}$$

### 2.4. Energy Expressions

The Lagrangian function of the FGM cylindrical double-walled shell with an internal structure system is constructed from the energy term for the strain, the kinetic energy term for the FGM shell/plate substructure, and the total elastic potential energy term of the FGM coupled structure, and its expression is

(43) $$\prod_{total}=\sum_{j=1}^{2}\left[\left(T_{cyj}-U_{cyj}-U_{s,cyj}\right)+\left(T_{rec,j}-U_{rec,j}\right)\right]+\sum_{i=1}^{N_{an}}\left(T_{ani}-U_{ani}-U_{s,ani}\right)+\prod_{F}-U_{total}$$

where *Π_F_* is the energy functional by the external force at the point of excitation, as

(44) $$\Pi_{F}=\iint_{S}\left(f_{u}u_{0}+f_{v}v_{0}+f_{w}w_{0}\right)dS$$

in which *f_x_*, *f_y,_* and *f_z_* is the external force which is applied in the *x*, *y,* and *z* directions. According to the Rayleigh–Ritz method, the dynamic model for vibration analysis of the FGM cylindrical double-walled shell with internal structure can be obtained, expressed as

(45) $$M\ddot{\Theta }+C\dot{\Theta }+K\Theta =F$$

where **M**, **C,** and **K** are, respectively, the mass, damping, and stiffness matrix for the FGM coupled structure system. **F** is the external load vector. **Θ** is the unknown coefficient vector with all expansions of unknown level expansion variables. For the free vibration solving of the FGM cylindrical double-walled shell with internal structure, the natural frequencies of the FGM cylindrical double-walled shell with internal structure under various boundary conditions can be easily solved by removing the external load vectors in Eq. Meanwhile, the forced vibration of the FGM cylindrical double-walled shell with internal structure can be obtained by solving the dynamics equation directly. With the modeling assumptions and computational approach established, we now present the results of the vibration analysis, including the natural frequencies and mode shapes for various configurations.

## 3. Numerical Results and Discussions

The vibration characteristics model of the FGM cylindrical double-walled shell with internal structure is established. In this part, the numerical investigation of the presented analysis model is proposed, including calculation verification, free vibration parameter investigation, and steady response parameter investigation. The geometric parameters of the presented model are set as *R*_1_ = 0.4 m, *R*_2_ = 0.8 m, *L* = 4 m, *N_an_* = 1, *x*_1_ = 0.5 *L*, *H*_1_ = *H*_2_ = *H_r_* = *h*_1_ = *h*_2_ = 0.01 m, *a*_1_ = 3 m, *a*_2_ = 1 m, *b*_2_ = 0.5 m, and *φ* = 60°. The material constants of FGM coupled structure are set as follows: (a) *E*_1_ = 168 GPa, *ρ*_1_ = 5700 kg/m^3^, *ν*_1_ = 0.3 for the zirconia; *E*_2_ = 70 GPa, *ρ*_2_ = 2707 kg/m^3^, and *ν*_2_ = 0.3 for the aluminum. The damping value and elastic supporting stiffness of the spring damping simulator are set as 10^14^ N/m and 1000 N/m∙s.

### 3.1. Calculation Verification

**Figure 6:**
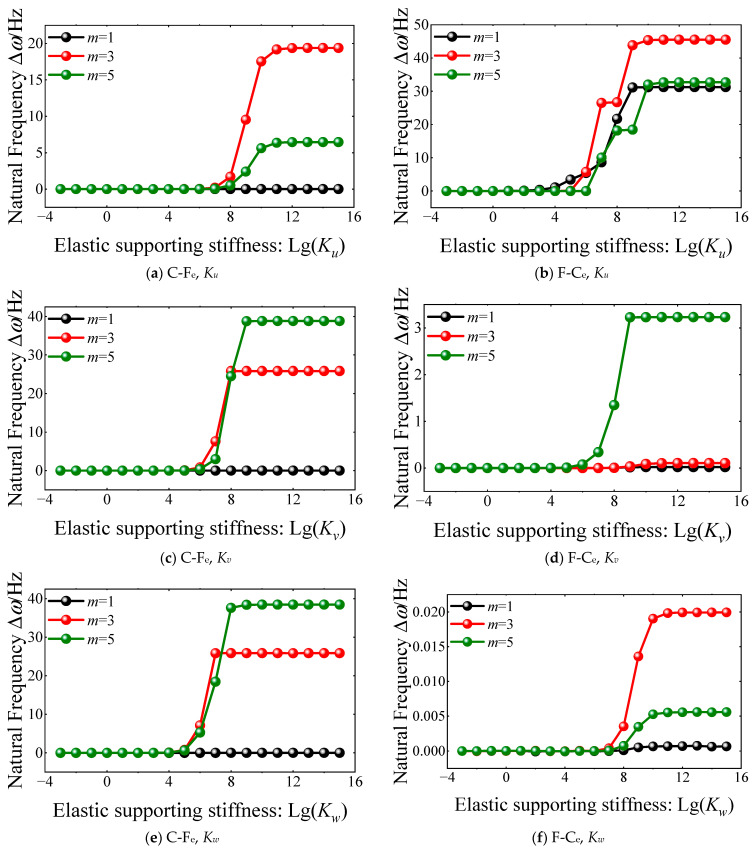
The variation of natural frequency ∆*ω* versus linear elastic spring stiffness for FGM cylindrical double-walled shell with internal structure with F-C_e_ boundary condition.

**Figure 7:**
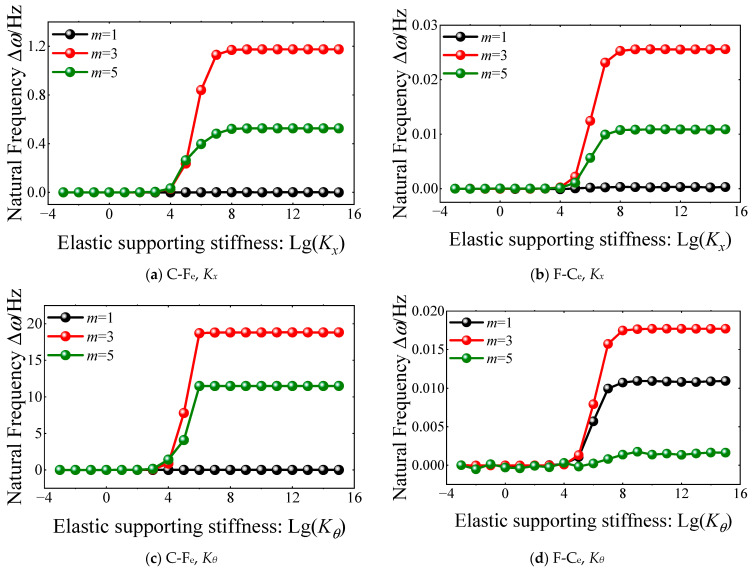
The variation of natural frequency ∆*ω* versus rotation elastic spring stiffness for FGM cylindrical double-walled shell with internal structure with C-F_e_ boundary condition.

The virtual boundary spring technology is adopted to achieve the boundary conditions for the FGM cylindrical double-walled shell with internal structure. Also, when the stiffness of the elastic boundary supporting spring stiffness is set to zero, the boundary condition of the FGM coupled structure is defined as free. Furthermore, when the value of the linear and rotation elastic boundary supporting spring stiffness is set to be infinite, the boundary condition is transformed as clamped. Then, in the calculation program of the vibration characteristics model for the FGM cylindrical double-walled shell with internal structure, the value of the spring stiffness cannot be set as infinite. So, Figure 6 and Figure 7 depict the influence of elastic spring stiffness on the natural frequencies of FGM cylindrical double-walled shells with internal structures with C-F*_e_* boundary conditions. It should be noted that boundary condition F*_e_* means that the linear and rotating elastic supporting stiffness is set as zero except for the changing boundary elastic supporting spring. From the variation curves of the natural frequencies in Figure 6, with the growth of the linear elastic spring stiffness *K_u_*, *K_v_*, and *K_w_*, there is an obvious growth trend for the natural frequencies. When the linear elastic spring stiffness is greater than 10^12^ N/m, the trend of each frequency order is gentle.

Also, for the variation curves of each order natural frequency of FGM coupled structure, the natural frequencies trend to gentle when the rotation elastic supporting stiffness is greater than 10^12^ N∙m/rad. So, the value of spring stiffness for the classical and elastic boundary conditions of the FGM cylindrical double-walled shell with internal structure in this article is shown in Table 1.

The spectral geometry method is selected to analyze the vibration characteristics of the FGM cylindrical double-walled shell with internal structure. In the admissible functions of the displacement variables, the value of *m* is infinite. For the mathematical model of the presented model, the value of *m* needs to be selected and determined. In Table 2, the first eight natural frequencies of FGM cylindrical double-walled shells with internal structures with C-C boundary conditions are shown. The truncation value for the cylindrical shell *M_cy_*, annular plate *M_an_*, base plate *M_rec_*_1,_ and vibration isolation plate *M_rec_*_2_ are selected, as *M_cy_* = *M_rec_*_1_ = 25, 28, 30, and 32, the value of *M_an_* × *M_rec_*_2_ is 16 × 30, 18 × 32, 20 × 35 and 21 × 36. It appears from the comparison that with the increase in the truncated value, the natural frequency gradually tends to be flat. Taking into account the efficiency of the calculation, in the subsequent calculation, *M_cy_* = *M_rec_*_1_ = 30, *M_an_* = 21, and *M_rec_*_2_ = 35.

Next, the calculation correctness of the presented model for FGM cylindrical double-walled shell with internal structure is verified. In Table 3, the comparison of the top ten natural frequencies of FGM cylindrical double-walled shell with internal structure by finite element method (FEM) and spectral geometry method (SGM) are listed. The boundary condition is set as C-C, and the material parameters of isotropic material are selected as *E*_1_ = *E*_2_ = 206 GPa, *ν*_1_ = *ν*_2_ = 0.3, and *ρ*_1_ = *ρ*_2_ = 7850 kg/m^3^. The error in Table 3 is set as (*ω*_SGM_ − *ω*_FEM_)/*ω*_SGM_. The FGM finite element model is built using Comsol analysis software. Among them, free triangular cells are used for meshing in the form of free division, and the cell size is set to 0.08 m. A total of 846 edge cells, as well as 13,064 boundary cells, are obtained. By comparison, the results obtained with different material settings are in good agreement, with the maximum error of 4.64%, located at the sixth-order natural frequency with the setting of isotropic material. Therefore, the correctness of the vibration characteristics model of the FGM cylindrical double-walled shell with internal structure for calculating the natural frequency can be proved.

**Figure 8:**
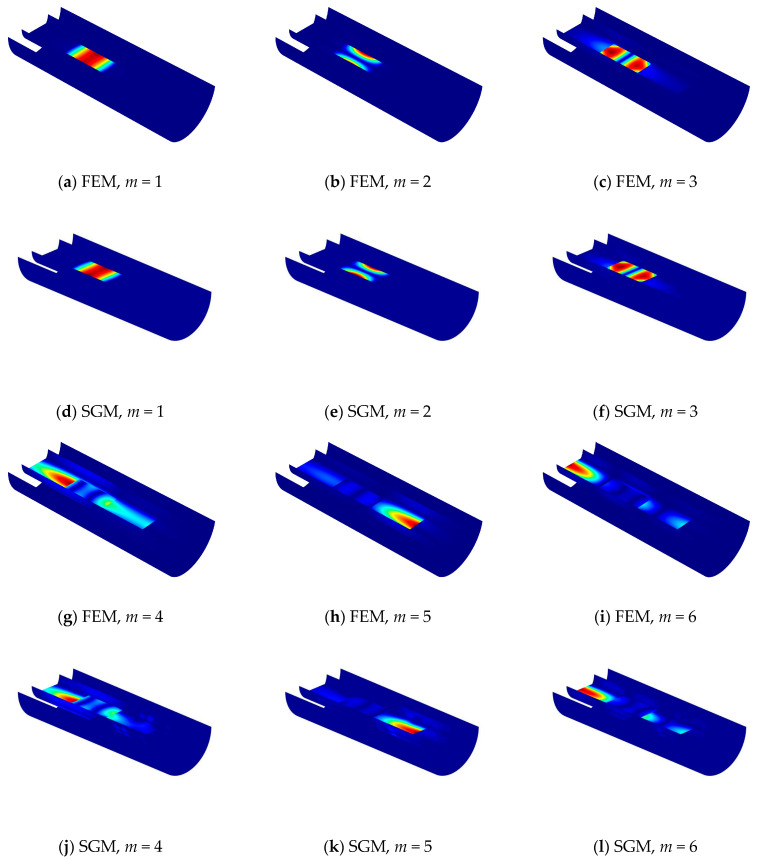
Comparison of the mode shapes for FGM cylindrical double-walled shell with internal structure by FEM and SGM with C-C boundary condition.

Then, to further verify the calculation correctness of the presented model of FGM coupled structure, the comparison of some selected mode shapes for FGM cylindrical double-walled shells with internal structure under the C-C boundary condition is displayed in Figure 8.

**Figure 9:**
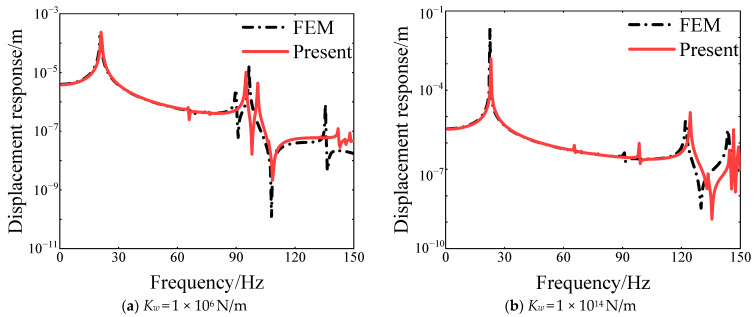
Comparison of displacement response of FGM cylindrical double-walled shell with internal structure under C-C boundary conditions with various elastic supporting stiffness *K_w_*.

Furthermore, the calculation correctness of the steady response for the vibration analysis model in this article is verified. In Figure 9, the comparison of displacement response of FGM cylindrical double-walled shell with internal structure under C-C boundary conditions with various elastic supporting stiffness *K_w_* is shown. It can be seen that the FEM response curves and the current analytical model are well aligned.

### 3.2. Investigations on Free Vibrations

**Figure 10:**
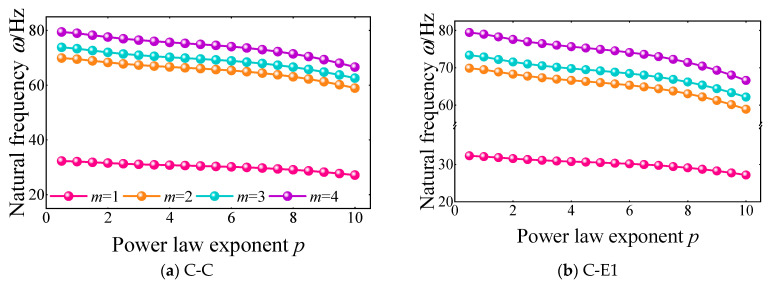
Variation of the first four-order natural frequency with power law exponent *p* under C-C and C-E1 boundary conditions of the FGM cylindrical double-walled shell with internal structure.

In this part, the influence of certain parameters on the free vibration characteristics of FGM cylindrical double-walled shells with internal structures with various boundary conditions is proposed. The natural frequencies of the FGM cylindrical double-walled shell with internal structure with the classical and elastic boundary conditions vary with several parameters shown, such as material constants, geometric parameters, and location variables. Figure 10 represents the variation of the first four orders on the natural frequencies of the FGM coupled structure with the power law exponent *p* at boundary conditions C-C and C-E1. It can be seen that the increase in the power law exponent *p* leads to significant attenuation of the first four orders’ natural frequencies. It can be found that as the frequency order increases, the effect of the power law exponent *p* on the attenuation of the natural frequency is gradually obvious. This indicates that an increase in the value of the power law exponent *p* leads to a gradual increase in the equivalent mass of the FGM structure, which in turn affects the change in natural frequencies under different conditions, causing them to gradually decrease.

**Figure 11:**
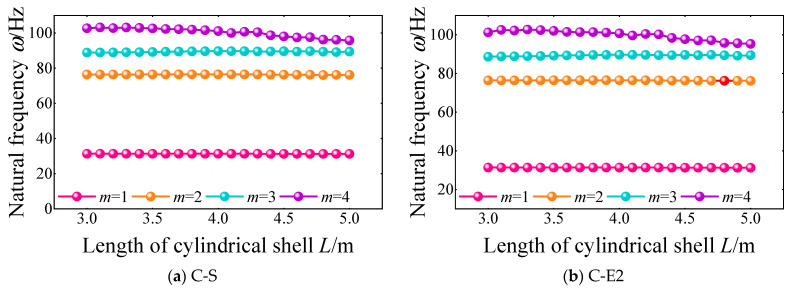
Variation of the first four-order natural frequency with the length of cylindrical shell *L* under C-S and C-E2 boundary conditions of the FGM cylindrical double-walled shell with internal structure.

Then, the effect of the length of the cylindrical shell on the natural frequencies of the FGM cylindrical double-walled shell with internal structure with different boundary conditions is proposed in Figure 11. The boundary condition is set as C-S and C-E2, and the length of cylindrical shell *L* is selected as 3–5 m. It can be seen that with the increase in the length of the cylindrical shell, the natural frequencies of the FGM double-layered coupled structure are generally decreased. Also, with the growth of the frequency order, the effect of the length of the cylindrical shell on the variation of the natural frequencies is more obvious.

**Figure 12:**
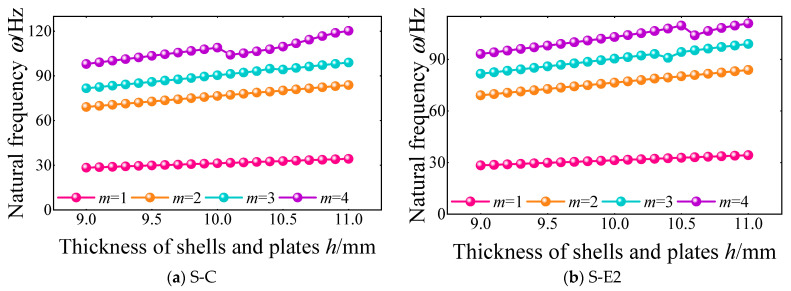
Variation of the first four-order natural frequency with the shell/plate thickness *h* under S-C and S-E2 boundary conditions of the FGM cylindrical double-walled shell with internal structure.

Next, the influence of the shell/plate thickness on the free vibration characteristics of the FGM cylindrical double-walled shell with internal structure with S-C and S-E2 boundary conditions is proposed. The variation curves of the first four natural frequencies are shown in Figure 12. In this numerical example, the thicknesses of the cylindrical shell *H*_1,2_, annular plate *H_r_*_,*i*_, base plate *h*_1,_ and vibration isolation plate *h*_2_ are set as *H*_1,2_ = *H_r_*_,*i*_ = *h*_1_ = *h*_2_ = *h*. When the shell/plate thickness increased from 9 mm to 11 mm, the first four natural frequencies of the FGM cylindrical double-walled shell with internal structure increased significantly. The breakage observed in mode number 4 suggests that the system’s response is highly sensitive to changes in the material gradient or boundary constraints at this particular mode. Also, the growth trend of the higher-order natural frequency is more obvious than the lower-order.

**Figure 13:**
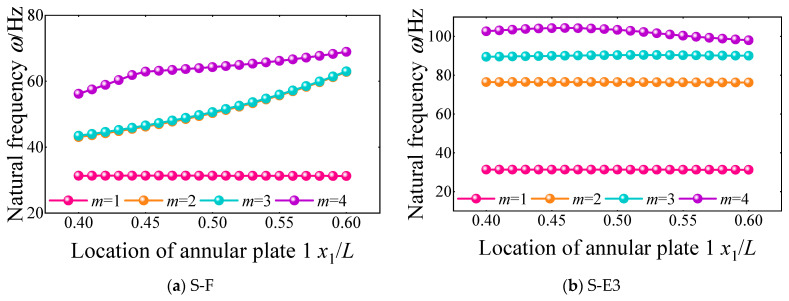
Variation of the first four-order natural frequency with the location of annular plate 1 *x*_1_/*L* under S-F and S-E3 boundary conditions of the FGM cylindrical double-walled shell with internal structure.

Then, the effect of the location of the annular plate on the natural frequencies of FGM cylindrical double-walled shells with internal structures with various boundary conditions is discussed in Figure 13. The boundary conditions of the FGM coupled structure are selected as S-F and S-E3. From the variation curves of the first four natural frequencies, when the location of the annular plate changes from 0.4 to 0.6, the natural frequencies of each order for the FGM coupled structure have a significant change phenomenon. When the boundary condition is S-F, the natural frequency shows a significant growth trend. At the same time, the growth trend of high-order frequency is more obvious than that of low-order frequency. When the boundary condition is S-E3, the changing trend of the natural frequency is not obvious, and the fourth-order natural frequency shows a significant attenuation trend.

### 3.3. Investigations on Steady-State Responses

**Figure 14:**
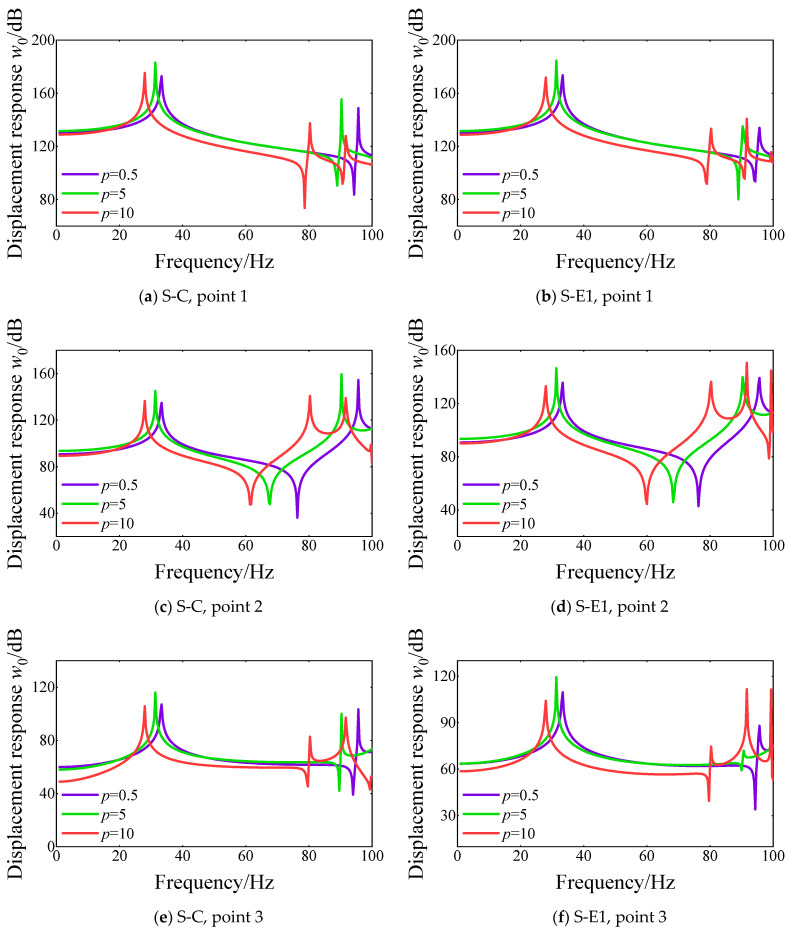

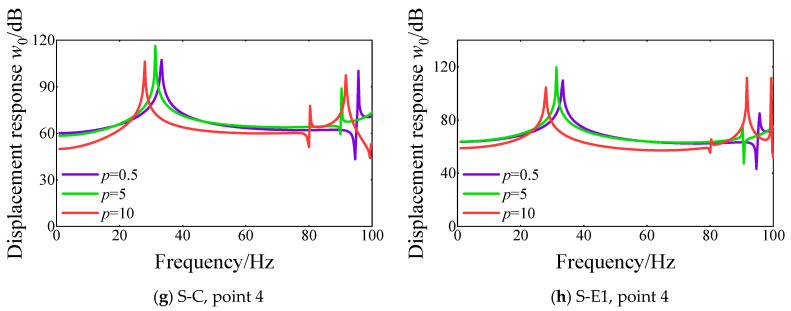
Comparison of displacement response curves for various power law exponent *p* of FGM cylindrical double-walled shell with internal structure at various response points under S-C and S-E1 boundary conditions.

Section 3.2 proposes the influence of several parameters on the natural frequencies of FGM cylindrical double-walled shells with internal structures with classical and elastic boundary conditions. In this part, the effect of some parameters on the steady response of FGM coupled structure with various boundary conditions, including classical and elastic boundary conditions, is discussed. The external application force is located on the vibration isolation plate at (0.3*a*_2_, 0.5*b*_2_) and (0.6*a*_2_, 0.5*b*_2_). The amplitude of the external force is 1 N. Also, there are four displacement response points of FGM coupled structure on cylindrical shell 1, cylindrical shell 2, the base rectangular plate, and the vibration isolation plate. The response point 1 is on the vibration isolation point as (0.5*a*_2_, 0.5*b*_2_), point 2 is on the base rectangular plate as (0.25*a*_1_, 0.5*b*_1_), point 3 is on cylindrical shell 1 as (0.5*L*, π/2), point 4 is on cylindrical shell 2 as (0.5*L*, π/2). In Figure 14, the displacement response curves of various displacement response points are shown at three different power law exponents, namely *p* = 0.5, 5, and 10. The boundary condition of the FGM coupled structure is selected as S-C and S-E1. It can be found that the power law exponent has a significant effect on the peak position of the displacement response curves of the FGM coupled structure with various boundary conditions. When the power law exponent changes from 0.5 to 10, the peak position of the displacement response curves gradually moves to the low-frequency direction. Furthermore, the effect of the power law exponent on the displacement response amplitude is more obvious on the displacement response curves of response points 3 and 4. When the power law exponent gradually increases, the displacement response amplitude attenuates significantly in the initial frequency range.

**Figure 15:**
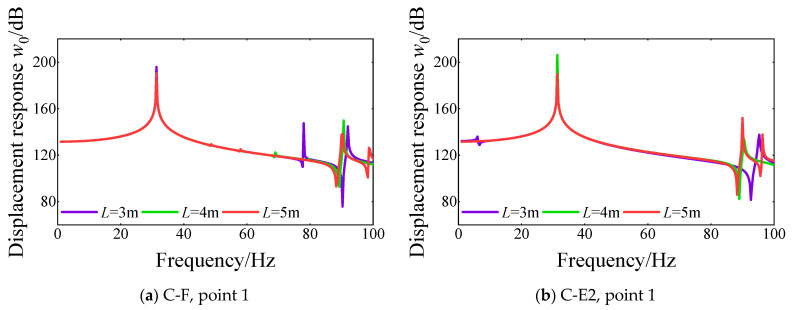

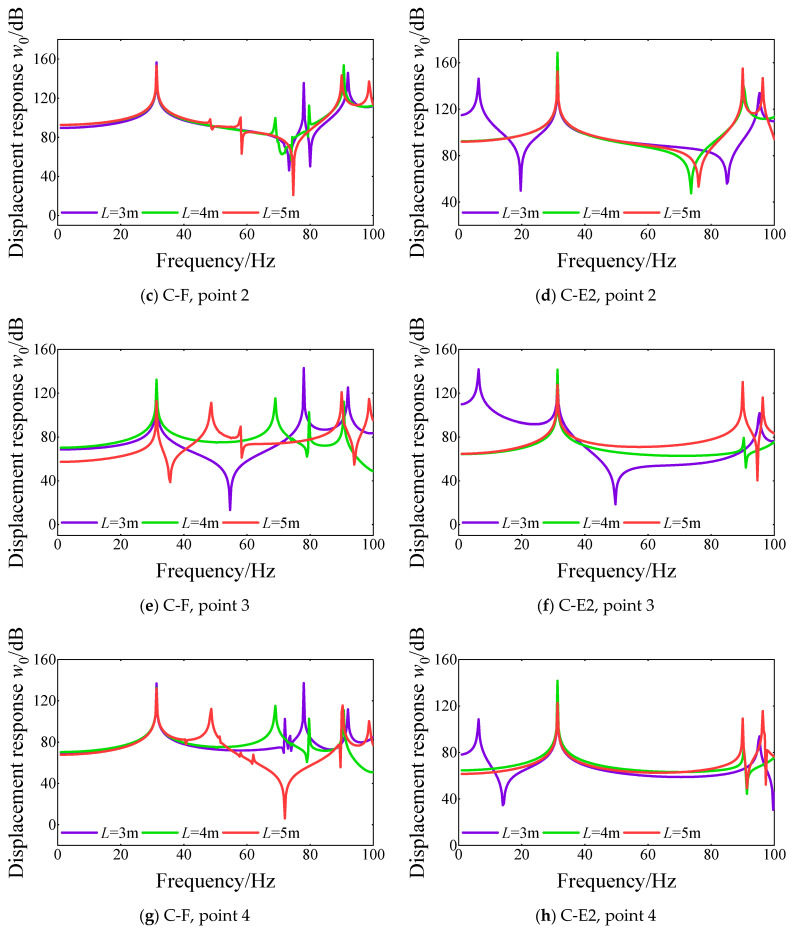
Comparison of displacement response curves for various lengths of cylindrical shell *L* of FGM cylindrical double-walled shell with internal structure at various response points under C-F and C-E2 boundary conditions.

Next, the influence of the length of the cylindrical shell on the steady response of FGM cylindrical double-walled shell with internal structure with various boundary conditions is investigated. The steady response curves of different response points for FGM coupled structures with various lengths of the cylindrical shell are shown in Figure 15 From the comparison of the displacement response curves, the length of the cylindrical shell has a significant effect on the peak location, with almost no significant effect on the amplitude of the displacement response. Also, with the increase in the cylindrical shell’s length, the displacement response curves’ peak location moves toward the low-frequency order. Further comparison shows that the effect of the length of the cylindrical shell is more obvious in the high-order frequency range than in the low-order frequency range.

**Figure 16:**
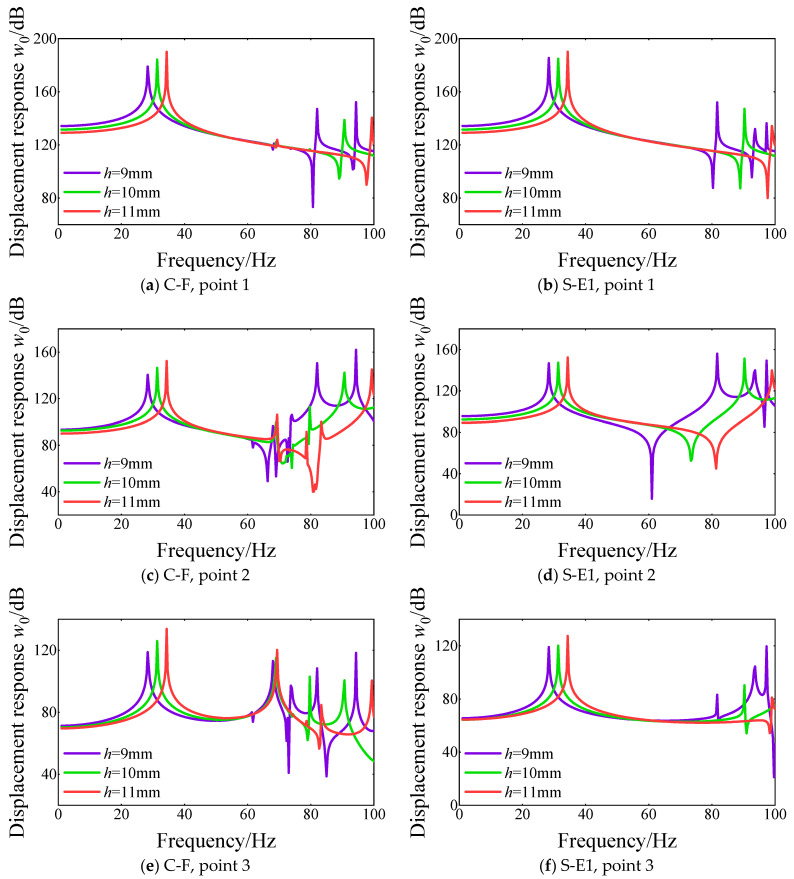

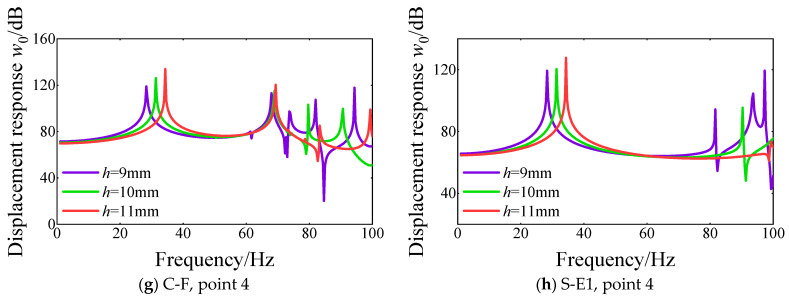
Comparison of displacement response curves for various lengths of shell/plate thickness *h* of FGM cylindrical double-walled shell with internal structure at various response points under C-F and S-E1 boundary conditions.

Then, the effect of shell/plate thickness on the steady response of FGM cylindrical double-walled shell with internal structure under various boundary conditions is proposed. The steady response curves at different response points versus shell/plate thickness are shown in Figure 16, namely *h* = 9 mm, 10 mm, and 11 mm. From the comparison of the displacement response curves at various displacement response points, the influence of the shell/plate thickness is obvious on the peak positions and displacement response amplitude. With the increase in the shell/plate thickness, the peak positions of the displacement response curves at different response points move toward the high-order frequency order. Also, the displacement response amplitude in the initial frequency order becomes significantly smaller as the shell/plate thickness increases.

**Figure 17:**
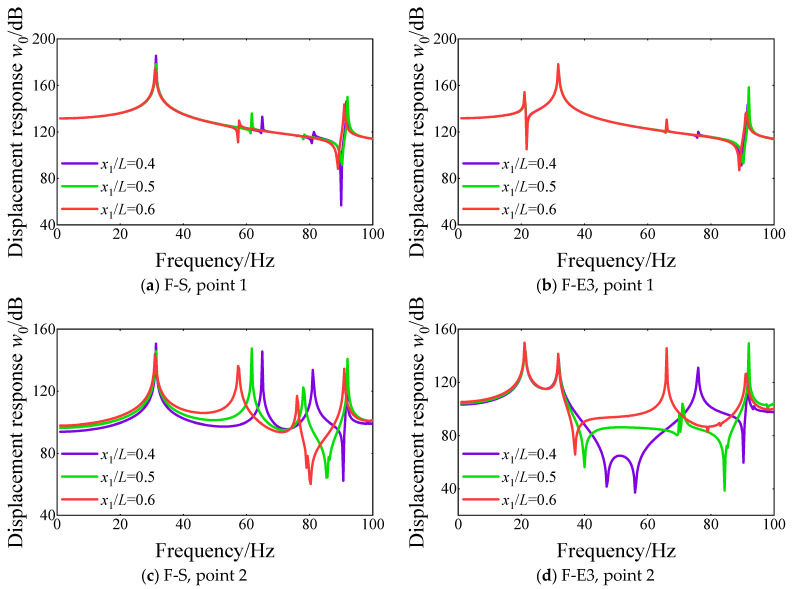

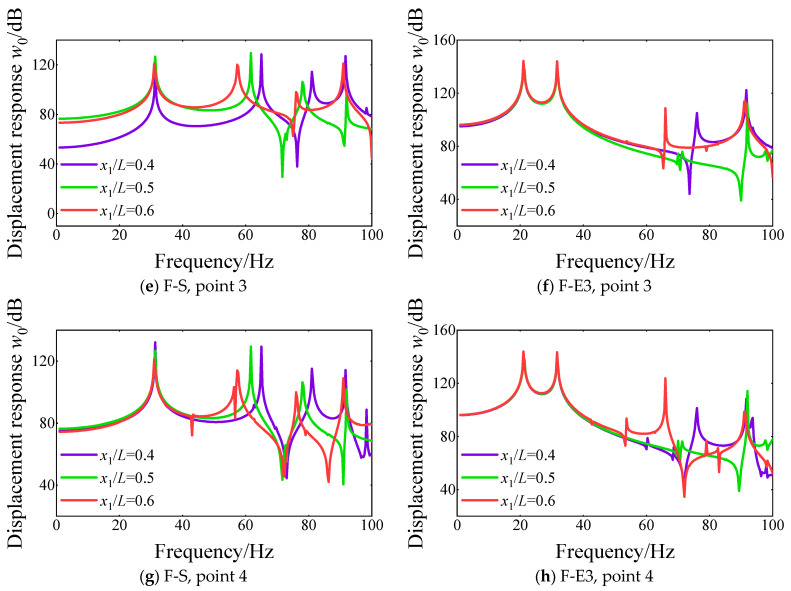
Comparison of displacement response curves for various locations of annular plate 1 *x*_1_/*L* of FGM cylindrical double-walled shell with internal structure at various response points under F-S and F-E3 boundary conditions.

Figure 17 shows the effect of the location of the annular plate on the steady response curves at different response points. The boundary condition of the FGM cylindrical double-walled shell with internal structure is set as F-S and F-E3. From the comparison of the displacement response curves at various boundary conditions, when the location of the annular plate changes from 0.4 to 0.6, the peak position of the displacement response curves moves toward the low-frequency direction. Furthermore, the consequences of the location of the annular plate on the peak position of displacement response curves in the high-frequency range are more obvious than in the low-frequency range.

**Figure 18:**
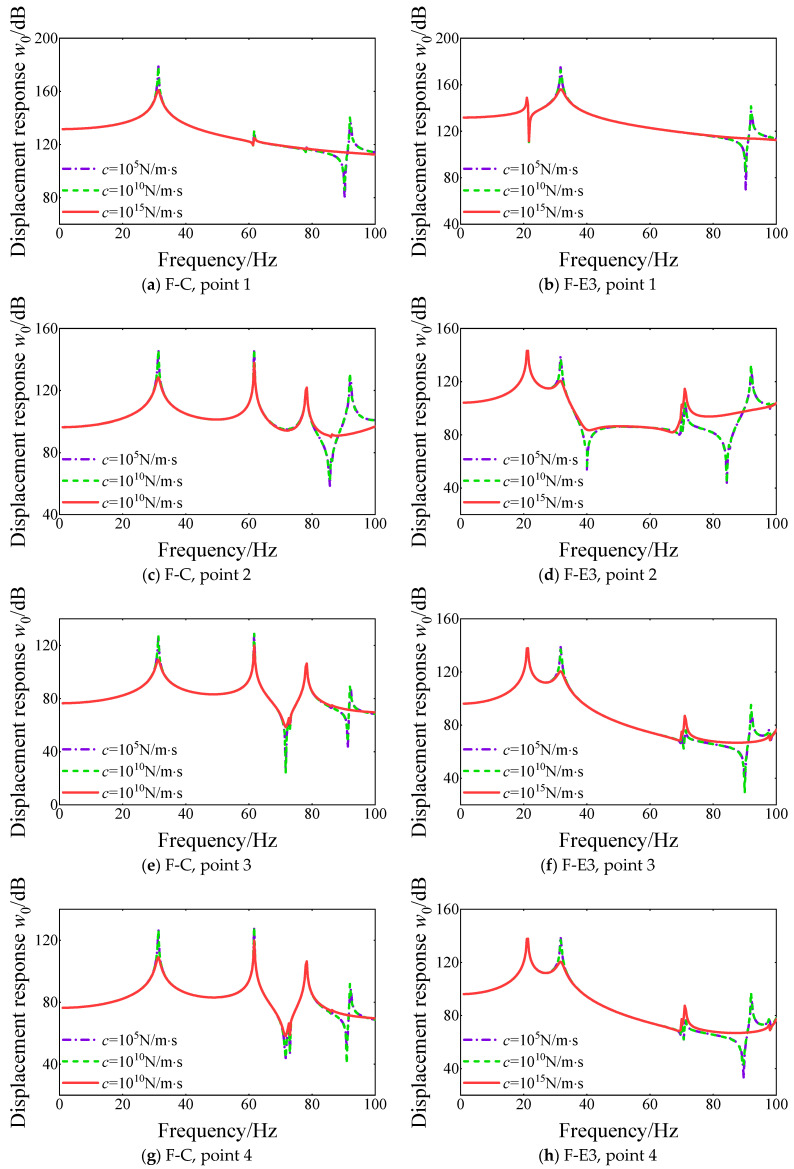
Comparison of displacement response curves for various damping values *c* of FGM cylindrical double-walled shell with internal structure at various response points under F-C and F-E3 boundary conditions.

Also, the influence of the damping value on the steady response curves of the FGM cylindrical double-walled shell with internal structure is proposed in this part. The displacement response curves at various response points versus different damping values are shown in Figure 18. It can be seen that the displacement response curves with various damping values for FGM cylindrical double-walled shells with internal structures with F-C and F-E3 boundary conditions have obvious changes. The peak position of the displacement response curves with various damping values remains unchanged. But, with the increasing damping value, some resonance peaks are missed in the high-frequency range. Also, with the increasing damping value, displacement response amplitude is significantly attenuated at the resonance peaks. The variation in damping does not have a significant impact on the overall equivalent stiffness and equivalent mass of the FGM coupled structure. As a result, it can be observed that the resonance peak positions of the curves remain largely unchanged. However, the maximum resonance peak value experiences noticeable attenuation, as the introduction and increase in damping factors lead to a gradual dissipation of energy. The results of the vibration analysis reveal significant insights into the dynamic behavior of FGM cylindrical shells, which we will now discuss in relation to the existing literature and their practical applications.

## 4. Conclusions

This study presents a comprehensive analysis of the dynamic behavior of functionally graded material (FGM) cylindrical double-walled shells, with a particular focus on vibration characteristics in underwater vehicle applications. By modeling FGM materials and performing numerical simulations, the following key findings were achieved:

1. The model successfully demonstrates how FGM material gradation and boundary conditions significantly influence the vibration characteristics of the structure;
2. The analysis highlights the potential of FGMs to enhance vibration damping, noise suppression, and overall performance in underwater vehicle applications;
3. Forced vibration analysis reveals that FGM materials improve structural stability and performance under dynamic loading conditions, suggesting their suitability for high-performance engineering applications.

Limitations include the assumption of perfect FGM materials, linear vibrations, and specific boundary conditions. Future research could explore the effects of material imperfections, nonlinear vibrations, and complex loading conditions. Additionally, expanding the model to include multi-physics simulations could further optimize FGM structures in practical applications.

In conclusion, this study establishes a solid foundation for the design and optimization of FGM cylindrical double-walled shells, providing valuable insights for both theoretical research and practical applications in underwater vehicles and other dynamic systems.

## Figures and Tables

**Table 1:** Spring stiffness value of boundary elastic supporting stiffness corresponding to the classical and elastic boundary conditions.

	*K_u_* (N/m)	*K_v_* (N/m)	*K_w_* (N/m)	*K_α_* (N∙m/rad)	*K_β_* (N∙m/rad)
Free (F)	0	0	0	0	0
Clamped (C)	1 × 10^14^	1 × 10^14^	1 × 10^14^	1 × 10^14^	1 × 10^14^
Simply supported (S)	1 × 10^14^	1 × 10^14^	1 × 10^14^	0	1 × 10^14^
Elastic_1 (E1)	1 × 10^8^	1 × 10^8^	1 × 10^14^	1 × 10^14^	1 × 10^14^
Elastic_2 (E2)	1 × 10^8^	1 × 10^8^	1 × 10^8^	1 × 10^14^	1 × 10^14^
Elastic_3 (E3)	1 × 10^8^	1 × 10^8^	1 × 10^8^	1 × 10^8^	1 × 10^14^

**Table 2:** Comparison of the natural frequency for the FGM cylindrical double-walled shell with internal structure with various truncation values *M_cy_*, *M_an_*, *M_rec_*_1,_ and *M_rec_*_2_.

*M_cy_*× *M_re_*_1_	*M_an_*× *M_rec_*_2_	*m*							
		1	2	3	4	5	6	7	8
25	16 × 30	30.247	74.997	88.739	100.877	105.762	112.295	126.239	132.496
	18 × 32	30.247	74.997	88.740	100.877	105.762	112.295	126.239	132.496
	20 × 35	30.247	74.997	88.740	100.877	105.762	112.295	126.239	132.496
	21 × 36	30.247	74.997	88.740	100.877	105.762	112.295	126.239	132.496
28	16 × 30	30.009	74.696	88.164	99.700	104.093	110.933	125.190	131.329
	18 × 32	30.009	74.696	88.164	99.700	104.093	110.933	125.190	131.329
	20 × 35	30.009	74.696	88.164	99.700	104.093	110.933	125.190	131.329
	21 × 36	30.009	74.696	88.164	99.700	104.093	110.933	125.190	131.329
30	16 × 30	29.831	74.441	87.990	99.409	103.650	110.580	124.749	131.090
	18 × 32	29.831	74.441	87.991	99.408	103.652	110.582	124.746	131.089
	20 × 35	29.830	74.441	87.992	99.409	103.652	110.582	124.747	131.088
	21 × 36	29.831	74.441	87.989	99.410	103.651	110.584	124.748	131.089
32	16 × 30	29.642	74.186	87.728	98.905	103.065	110.032	124.254	130.600
	18 × 32	29.642	74.186	87.728	98.905	103.065	110.032	124.254	130.599
	20 × 35	29.642	74.186	87.728	98.905	103.065	110.032	124.254	130.599
	21 × 36	29.642	74.186	87.728	98.905	103.065	110.032	124.254	130.599

**Table 3:** Comparison of top ten natural frequencies of FGM cylindrical double-walled shell with internal structure under C-C boundary condition with various material types.

Mode	Functionally Graded Material			Isotropic Material		
	FEM	SGM	Error	FEM	SGM	Error
1	22.572	23.1835	2.64%	22.759	23.2234	2.00%
2	66.114	67.76595	2.44%	66.643	67.8521	1.78%
3	77.241	79.73345	3.13%	77.813	79.8064	2.50%
4	94.655	97.12825	2.55%	94.963	96.9137	2.01%
5	102.11	101.8935	−0.21%	102.59	101.6498	−0.92%
6	113.55	109.7987	−3.42%	114.19	109.1312	−4.64%
7	121.3	121.4472	0.12%	122.06	121.4790	−0.48%
8	127.18	126.2301	−0.75%	127.97	126.7965	−0.93%
9	130.06	131.1448	0.83%	127.97	130.1468	1.67%
10	130.06	132.7009	1.99%	128.10	130.1502	1.58%

## Data Availability

The original contributions presented in this study are included in the article. Further inquiries can be directed to the corresponding author.
